# Convergent validity of taekwondo high-intensity intermittent sport-specific tests and their relationship with lower limb muscle power performance

**DOI:** 10.3389/fphys.2026.1825858

**Published:** 2026-06-01

**Authors:** Gennaro Apollaro, Emerson Franchini, Rafael L. Kons, Chris Bishop, Marco Panascì, Piero Ruggeri, Coral Falcó, Emanuela Faelli

**Affiliations:** 1 Department of Neuroscience, Rehabilitation, Ophthalmology, Genetics and Maternal Child Health, University of Genoa, Genoa, Italy; 2 Centro Polifunzionale di Scienze Motorie, Università degli Studi di Genova, Genoa, Italy; 3 Martial Arts and Combat Sports Research Group, Sport Department, School of Physical Education and Sport, University of São Paulo, São Paulo, Brazil; 4 Department of Physical Education, Faculty of Education, Federal University of Bahia, Bahia, Brazil; 5 London Sport Institute, Faculty of Science and Technology, Middlesex University, London, United Kingdom; 6 Department of Experimental Medicine, Section of Human Physiology, University of Genoa, Genoa, Italy; 7 Department of Sport, Food and Natural Sciences, Western Norway University of Applied Sciences, Bergen, Norway

**Keywords:** anaerobic, assessment, combat sports, Olympic sports, physical tests

## Abstract

**Objective:**

The interchangeability between multiple Frequency Speed of Kick Test (FSKT_mult_) and chest Taekwondo Anaerobic Intermittent Kick Test (TAIKT_chest_) has not been established, which prevents determining whether they reflect the same underlying construct of ability to repeat high-intensity kicks. Moreover, the relationship between the ability to repeat high-intensity kicks and lower limb muscle power requires further investigation. This study aimed to: establish the construct convergent validity between the FSKT_mult_ and TAIKT_chest_; investigate their relationship with countermovement jump (CMJ) test.

**Methods:**

Twenty-two national/international-level taekwondo athletes participated in this study. On the first day, they performed three CMJ followed by one of the intermittent tests in a randomized and counterbalanced order. After 48 hours, the other intermittent test was performed. During the FSKT_mult_ and TAIKT_chest_, heart rate (HR) was measured, while blood lactate concentration ([La]) and rating of perceived exertion (RPE) were recorded at the end of the tests.

**Results:**

FSKT_mult_ total kick performance, as well as absolute and relative power performances, showed high shared variance (*R*^2^ = 62–92%, *p* < 0.001) with respective TAIKT_chest_ performances. HR, [La], and RPE responses were significantly higher (*p* ≤ 0.001, Cohen’s *d* = 0.4–1.3) in the FSKT_mult_ than in TAIKT_chest_. Most high-intensity intermittent performances showed high significant correlations (*r* = 0.51–0.78, *p* < 0.05) with respective CMJ performances.

**Conclusions:**

FSKT_mult_ performances can be predicted from the respective TAIKT_chest_ performances and vice versa. However, the distinct methodological characteristics of the tests generate specific performance, physiological, and perceptual dynamics. Lower limb muscle power could influence performance in high-intensity kicks.

## 1 Introduction

The majority of scientific research focused on specific assessment for Olympic combat sports (i.e., boxing, fencing, judo, taekwondo, and wrestling) has been conducted over the last two decades (Chaabene et al., 2018; Kons et al., 2023; Apollaro et al., 2024a; Apollaro et al., 2024b). A systematic review on the methodological quality, validation data, and feasibility of tests designed to better reproduce the physical and physiological demands of these Olympic disciplines (Chaabene et al., 2018), concluded that the research body produced up to 2017 could be considered in its intermediate stage of development, as it is characterized by not fully established theories and several methodological shortcomings (Edmondson and McManus, 2007). By critically analyzing the literature, the authors found that construct validity received less attention than translational and criterion validity, representing one of the main shortcomings of the validation data (31%, 98% and 95% of studies, respectively) (Chaabene et al., 2018). In this regard, all types of validity are important in the assessment of physical qualities, and it is also necessary to provide evidence for each of their different subdomains (Weakley et al., 2024). In particular, the convergent side of construct validity (i.e., the extent to which a sport-specific test and another measure of the same construct are indeed related (Weakley et al., 2024)) was not investigated in any of the studies examined (Chaabene et al., 2018). Consequently, although the new sport-specific tests may be more ecological, it is still unclear whether they measure the same construct that is intended to be assessed. Thus, the study of convergent validity emerges as an important step in the ongoing development of this field.

Subsequent narrative reviews conducted to provide practical applications, recommendations, and future perspectives as guidelines for sport-specific assessment in taekwondo have suggested a particular focus on monitoring the fundamental fitness components of this sport from a more ecological, simple, and non-invasive perspective (Apollaro et al., 2024a; Apollaro et al., 2024b). In particular, several sport-specific tests from a methodological and measurement point of view have been validated to assess anaerobic fitness. The focus on this component is justified by the fundamental role of anaerobic energy systems (Apollaro et al., 2024b) in supporting the short (~2 seconds [s]) and intermittent (attack/skipping [A/S] ratio between ~1:1.3 and 1:8) attack activity of combat (Bridge et al., 2011; Santos et al., 2011; Bridge et al., 2013; Apollaro et al., 2023; Apollaro et al., 2025). Among the specific protocols implemented, the multiple Frequency Speed of Kick Test (FSKT_mult_) and the chest Taekwondo Anaerobic Intermittent Kick Test (TAIKT_chest_) are the most studied and used tests in practice to assess high-intensity intermittent performance (Tayech et al., 2019; da Silva Santos et al., 2020; Apollaro et al., 2024b). The FSKT_mult_ has translational logical validity, construct discriminant validity, sensitivity, test-retest and intra-/inter-rater reliability (Apollaro et al., 2024b), and its inability to predict the temporal structure of the match has recently been documented (Apollaro et al., 2025). Instead, the TAIKT_chest_ has translational content validity, criterion concurrent validity, construct discriminant validity, and test-retest reliability (Apollaro et al., 2024b).

The FSKT_mult_ and TAIKT_chest_ share the same kicking technique (i.e., bandal-chagi to the trunk) and are both characterized by short all-out attack sets, short total test duration, and an intermittent structure. However, except for the kicking technique, these methodological characteristics differ between the two tests, namely in the duration of the attack sets, total test duration, and A/S ratio (i.e., 10 s vs. 5 s, 90 s vs. 80 s, and 1:1 vs. 1:2, respectively) (Tayech et al., 2019; da Silva Santos et al., 2020; Apollaro et al., 2024b). In addition, the two tests have specific measurement characteristics. Specifically, the FSKT_mult_ performance parameters are based on the number of kicks applied to a traditional taekwondo body protector; thus, valid techniques are counted by one or more evaluators through a simple post-test video analysis procedure (da Silva Santos et al., 2020). In contrast, the TAIKT_chest_ performance parameters are expressed in terms of power using a specifically developed calculation method (which considers the projection distance of the foot and the mass of the lower limb), and valid kicks are automatically recorded by an electronic body protector; thus, eliminating the task of the evaluator and the resulting intra-/inter-rater reliability procedure typical of the FSKT_mult_ (Tayech et al., 2019). It is relevant to highlight that the use of a traditional body protector also for TAIKT_chest_ could have a positive impact on the cost of assessment and the applicability of the test, as the electronic scoring system is not available in all environments. Meanwhile, the method for expressing performance in terms of power could also be extended to FSKT_mult_, increasing the information that can be obtained from the test (Apollaro et al., 2024c). In this context, the availability of two widely studied and applied sport-specific tests for measuring the same construct, with similar but distinct methodological characteristics and shareable measurement characteristics, indicates the study of convergent validity as a fundamental step for establishing the interchangeability of protocols (Tayech et al., 2022).

In parallel, the availability of two intermittent sport-specific tests allows us to expand the study of the relationship between the ability to repeat high-intensity kicks and the lower limb muscle power, commonly assessed in taekwondo athletes through the countermovement jump (CMJ) test (da Silva Santos et al., 2018; Tayech et al., 2020; Albuquerque et al., 2022; Apollaro et al., 2024c). Some studies have found that the height reached in the CMJ explained between 17 and 26% of the variance of the total number of kicks in the FSKT_mult_ (Albuquerque et al., 2022; Apollaro et al., 2024c). On the other hand, a single study has found that the relative peak power of the CMJ explained 40% of the variance of the relative peak power of the TAIKT_chest_ (Tayech et al., 2020). Thus, the available evidence from sport-specific testing protocols (Tayech et al., 2020; Albuquerque et al., 2022; Apollaro et al., 2024c) suggests only moderate relationships between lower limb muscle power and the ability to repeat high-intensity kicks. At the same time, previous studies have consistently reported relatively low lower limb muscle power values in taekwondo athletes (Bridge et al., 2014; Cardozo and Moreno-Jiménez, 2018). Taken together, these findings indicate that, despite its potential relevance, the role of lower limb muscle power in repeated kicking performance is not yet fully understood, thus warranting further investigation into the relationship between these two fundamental requirements of competition.

Therefore, the first aim of this study was to establish the convergent validity between the FSKT_mult_ and TAIKT_chest_. It was hypothesized that the presence of positive and significant correlations between the variables of the tests as they were developed to assess the same construct in an intermittent and sport-specific setting. At the same time, it was hypothesized significantly different performances and physiological and perceptual responses between tests due to their distinct methodological characteristics, such as the duration of each set and of the entire test, as well as the A/S ratio. The second aim was to investigate the relationship between sport-specific tests (i.e., FSKT_mult_ and TAIKT_chest_) and lower limb muscle power test (i.e., CMJ). Positive and significant correlations were hypothesized between high-intensity intermittent performances and lower limb muscle power performances, with specific correlation patterns depending on the particular duration of the attack phases and A/S ratio of the intermittent activity.

## 2 Materials and methods

### 2.1 Study design

This cross-sectional observational study was conducted during the mid-phase of the competitive season when athletes were regularly engaged in national (i.e., national championships and cups) and international (i.e., G-1/E-1 and/or E-2 rank tournaments, and/or European championships) competitions. The testing sessions were conducted on two non-consecutive days. The high-intensity intermittent tests were performed in a randomized and counterbalanced order. On the first day, the CMJ test and FSKT_mult_ (or TAIKT_chest_) were performed, respectively, in the order established based on intensity. After 48 hours, the athletes performed the TAIKT_chest_ (or FSKT_mult_).

### 2.2 Participants

Based on data from Tayech et al (Tayech et al., 2020; Tayech et al., 2022), an *a priori* power analysis using G*Power software (v. 3.1.9.7; Heinrich Heine University in Düsseldorf, Germany) indicated that a total sample of 19 subjects would be required with the following parameters: bivariate normal model test (two-tailed), r = 0.60, α = 0.05, 1-β = 0.80. Twenty-two taekwondo black belt athletes (11 males and 11 females) participated in this study. The characteristics of athletes are detailed in **Table 1**. Inclusion criteria were: aged 15 years or older; more than 5 years of experience in taekwondo; be training at least 5 times a week; not engage in any acute rapid weight loss strategies during the study period; not having taken drugs, medications or dietary supplements; not having suffered muscle and joint injuries in the past 6 months. Exclusion criteria included the presence of pain, injury, or illness at the time of testing. No participants met the exclusion criteria. All athletes were recruited from the same local club, to prevent potential interference/variation induced by training programs’ variation, and they were following a standard training program of 8 weekly sessions (∼90 minutes/session) at the time of the study. Athletes provided a written informed consent form after they were informed about the design of the study and the possible benefits and risks associated with it. For athletes under the age of 18, written informed consent was obtained from their parents. The study was approved by the Local Ethics Committee (University of Genoa—approval no: 2024/44) and was conducted according to the Declaration of Helsinki (World Medical Association, 2013).

**Table 1:** Characteristics of athletes participating in the study.

Characteristics	Total (n = 22)	Male (n = 11)	Female (n = 11)
	M ± SD [Min–Max]	M ± SD [Min–Max]	M ± SD [Min–Max]
Age (year)	17.4 ± 2.0 [15–22]	17.2 ± 1.3 [15–20]	17.5 ± 2.6 [15–22]
Experience (year)	12.0 ± 2.2 [8–18]	11.4 ± 1.7 [9–15]	12.5 ± 2.6 [8–18]
Body height (cm)	170.6 ± 6.1 [157–181]	172.5 ± 6.0 [163–181]	168.7 ± 5.9 [157–176]
Body mass (kg)	57.3 ± 8.1 [45.1–73.6]	57.3 ± 7.0 [46.6–68.6]	57.2 ± 9.4 [45.1–73.6]
Body fat (%)	15.0 ± 9.6 [3–34.4]	6.5 ± 2.3 [3–10.9]	23.5 ± 5.6 [14.2–34.4]

M, mean; SD, standard deviation; Min, minimum; Max, maximum.

### 2.3 Procedures

**Figure 1:**
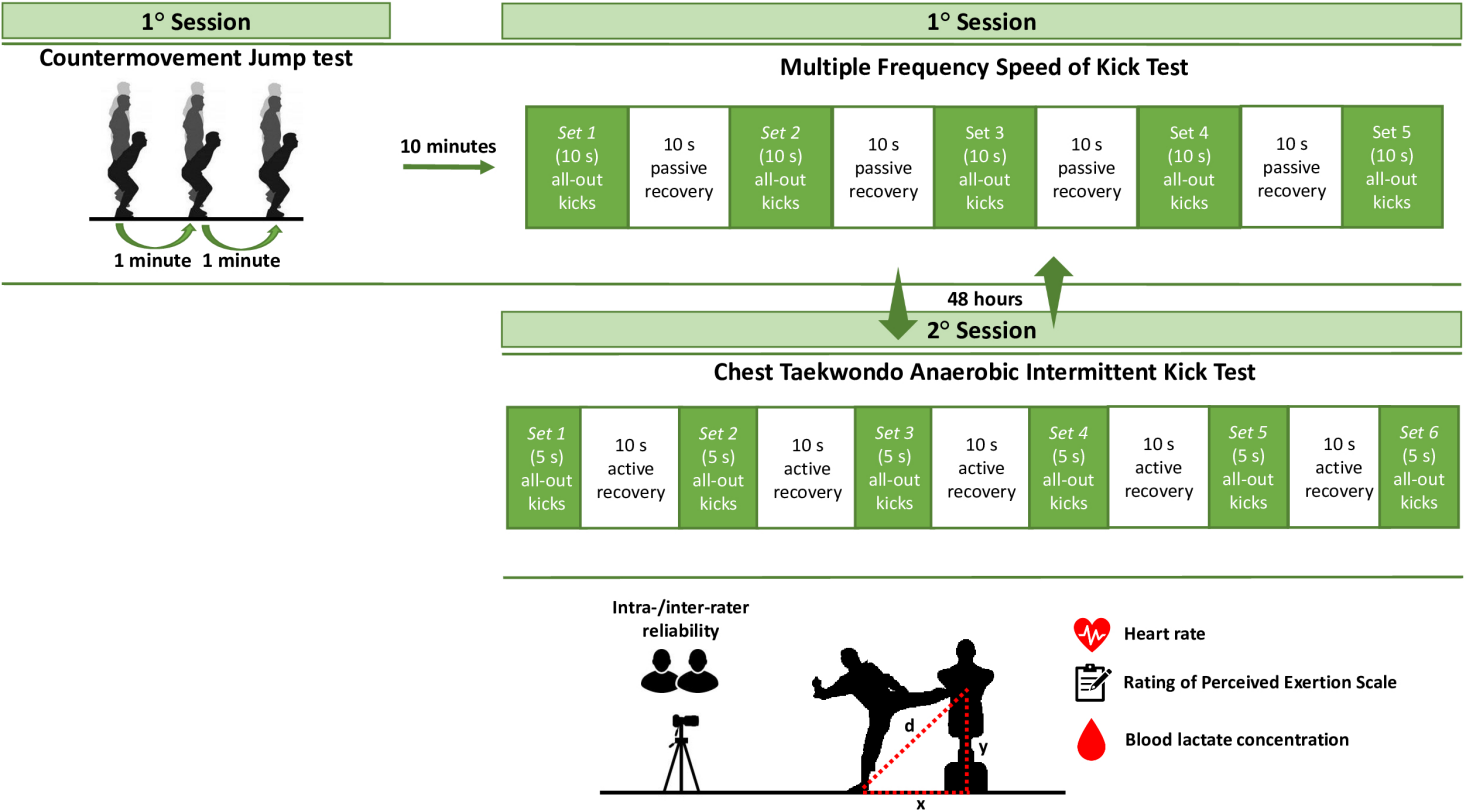
Schematic representation of the study design. s, second.

During the week preceding the evaluations, athletes were familiarized twice with all the procedures to minimize the learning effect (Chaabene et al., 2018). On these occasions, body height and composition were measured with a stadiometer (0.1 cm resolution; Seca Model 217; SECA GmbH & Co. KG., Hamburg, Germany) and a bioelectrical impedance scale (0.1 kg resolution; Tanita BC-420 MA; Tanita Corp., Tokyo, Japan), respectively. In the 24 hours before the two testing sessions, athletes were asked to avoid any strenuous physical activity, consumption of caffeine, energy drinks, and alcohol. In addition, athletes performed the testing sessions without consuming food for 2 hours beforehand. Both testing experimental sessions were conducted by the main researcher at the athletes’ sports center, at the same time of day (5:00–7:00 p.m.) and under similar temperature (22–25 °C) and humidity conditions (42–45%) to avoid any diurnal variation in performance. Prior to the sessions, athletes performed a general and specific warm-up routine consisting of running, stretching, kicking, and punching at low intensity for a total of 15 minutes (da Silva Santos et al., 2020; Tayech et al., 2022). After 5 minutes of passive recovery, the athletes began the evaluations. In the first testing session, three attempts of CMJ test were allowed and a passive recovery interval of 1 minute was applied between each attempt. After 10 minutes of passive recovery, athletes performed the FSKT_mult_ (or TAIKT_chest_). In the second session, athletes performed the TAIKT_chest_ (or FSKT_mult_). Heart rate (HR), blood lactate concentration ([La]), and rating of perceived exertion (RPE) were assessed for the high-intensity intermittent tests. The researcher consistently provided standard verbal encouragement to all athletes. The experimental procedures are detailed in **Figure 1**.

#### 2.3.1 Muscle power test

**Countermovement Jump**. Athletes performed the CMJ positioned with both feet on top of an electronic contact mat (Globus Ergo Jump; Globus Inc., Codognè, Italy) and their hands on their hips. They were asked to squat rapidly up to a self-determined squat depth, jump as high as possible (keeping their lower limbs extended during the aerial phase) and land on the contact mat (da Silva Santos et al., 2018; Apollaro et al., 2024c). Three attempts were allowed with 1 minute of passive recovery between them and the mean performance of the CMJ test was used for the subsequent statistical analysis, as previously suggested (Claudino et al., 2017) (within-session reliability: intraclass correlation coefficient (ICC) = 0.993, 95% confidence intervals (CI) = 0.986–0.997, excellent, p < 0.001; coefficient of variation (CV) = 3.0%, excellent; standard error of measurement (SEM) = 0.63).

**Performance**. Jump height (cm) was calculated by flight time using the Bosco’s method (9.81 × flight time^2^/8) (Bosco et al., 1983). Thus, CMJ height and the athletes’ body mass were used to calculate absolute (W) peak power (P_peak_), by Equation of Sayers et al. (1999) (60.7 × jump height (in cm) + 45.3 × BM (in kg) − 2055) and relative (W·kg^-0.67^) peak power, using allometric scaling (Tayech et al., 2020; Tayech et al., 2022), as follows: relative peak power = absolute peak power (W)/kg^0.67^. Furthermore, the CMJ net impulse (N.s) was calculated as reported by Wells et al. (2024): BM (in kg) × √[jump height (in m) × (2 × 9.81)].

#### 2.3.2 High-intensity intermittent sport-specific tests

***Multiple Frequency Speed of Kick Test.*** The FSKT_mult_ consists of five 10 seconds sets with a 10 seconds passive recovery between sets (da Silva Santos et al., 2020; Apollaro et al., 2024b). Each athlete was placed in front of the training dummy (bob-type punching dummy) equipped with a traditional taekwondo body protector, positioned in same height of the athlete trunk at a height (*y*) relative to the mat. During the kick, the athlete was not allowed to cross a mark on the mat in order to execute the kick effectively from an ideal distance (x) relative to the dummy (Tayech et al., 2019). After the sound signal emitted by the Timer Plus app (*v. 1.4.7; VGFIT LLC*), athletes executed the maximum number of bandal-chagi (i.e., roundhouse kicks) possible, alternating right and left legs (**Figure 1**).

**Chest Taekwondo Anaerobic Intermittent Kick Test**. The TAIKT_chest_ consists of six 5 seconds sets with a 10 seconds active recovery (i.e., very light stepping movements) between sets (Tayech et al., 2019; Apollaro et al., 2024b). The execution criteria for the TAIKT_chest_ are the same as those defined for the FSKT_mult_ (**Figure 1**).

**Video Analysis**. FSKT_mult_ and TAIKT_chest_ were recorded using a standard smartphone camera (iPhone 14; Apple Inc., Cupertino, USA). The videos were subsequently analyzed within the *Kinovea* software (v. 0.9.5; Joan Charmant and Contributors, Bordeaux, France) to manually count the valid kicks, in frame-by-frame mode with an accuracy of 0.03 s. First, the count of a kick started when the athlete moved the attack foot and finished when he/she touched the dummy. Kicks considered were those that hit the target during the designated time. If the athlete started the kick before completing the last second but reached the target only after it, the kick was not considered. Second, valid kicks were those performed with appropriate technique and power (da Silva Santos et al., 2020; Apollaro et al., 2024b). In agreement with the literature (Apollaro et al., 2024b), the main researcher (i.e., a taekwondo coach, ≥20 years of taekwondo experience and black belt) quantified the valid kicks twice, by separating each observation by a 7-day interval, to verify the intra-rater reliability. In parallel, a second researcher (i.e., a taekwondo coach, ≥30 years of taekwondo experience and black belt) quantified the valid kicks to establish the inter-rater reliability.

**Performance**. The performance was determined by the total number of kicks (n° kicks) and kick decrement index (KDI) calculated using the following equation: KDI (%) = [1 − (set_1_ + set_2_ + set_3_ + …)/best set × number of sets] × 100 (da Silva Santos et al., 2020; Apollaro et al., 2024b). **Table 2** reports the intra-/inter-rater reliability analyses of the FSKT_mult_ and TAIKT_chest_.

**Table 2:** Intra-/inter-rater reliability of the multiple frequency speed of kick test (FSKT_mult_) and chest taekwondo anaerobic intermittent kick test (TAIKT_chest_) performances (n = 22).

	Intra-rater reliability					Inter-rater reliability				
	Evaluator 1					Evaluator 1 and Evaluator 2				
FSKT_mult_	ICC [95%CI]	Magnitude	CV%	Magnitude	SEM	ICC [95%CI]	Magnitude	CV%	Magnitude	SEM
FSKT_1_ (n° kicks)	0.98 [0.96–0.99]*	excellent	0.52	excellent	0.18	0.99 [0.97–1.00]*	excellent	0.36	excellent	0.15
FSKT_2_ (n° kicks)	0.99 [0.97–0.99]*	excellent	0.50	excellent	0.18	0.98 [0.95–0.99]*	excellent	0.67	excellent	0.21
FSKT_3_ (n° kicks)	0.98 [0.96–0.99]*	excellent	0.38	excellent	0.15	0.99 [0.98–1.00]*	excellent	0.19	excellent	0.10
FSKT_4_ (n° kicks)	1.00 [1.00–1.00]*	excellent	0.00	excellent	0.00	0.99 [0.98–1.00]*	excellent	0.21	excellent	0.11
FSKT_5_ (n° kicks)	0.98 [0.96–0.99]*	excellent	0.40	excellent	0.15	0.98 [0.96–0.99]*	excellent	0.38	excellent	0.15
FSKT_BEST_ (n° kicks)	0.98 [0.96–0.99]*	excellent	0.52	excellent	0.18	0.99 [0.97–1.00]*	excellent	0.36	excellent	0.15
FSKT_TOTAL_ (n° kicks)	1.00 [0.99–1.00]*	excellent	0.37	excellent	0.38	1.00 [0.99–1.00]*	excellent	0.29	excellent	0.27
KDI (%)	0.89 [0.77–0.95]*	good	9.75	very good	0.98	0.94 [0.86–0.98]*	excellent	10.76	acceptable	0.66
TAIKT_chest_										
TAIKT_1_ (n° kicks)	1.00 [1.00–1.00]*	excellent	0.00	excellent	0.00	0.99 [0.97–0.99]*	excellent	0.34	excellent	0.11
TAIKT_2_ (n° kicks)	0.98 [0.95–0.99]*	excellent	0.28	excellent	0.11	0.96 [0.91–0.98]*	excellent	0.59	excellent	0.15
TAIKT_3_ (n° kicks)	0.96 [0.91–0.99]*	excellent	0.62	excellent	0.15	0.96 [0.91–0.99]*	excellent	0.62	excellent	0.15
TAIKT_4_ (n° kicks)	0.97 [0.92–0.99]*	excellent	0.62	excellent	0.15	0.98 [0.96–0.99]*	excellent	0.38	excellent	0.11
TAIKT_5_ (n° kicks)	0.97 [0.93–0.99]*	excellent	0.68	excellent	0.15	0.96 [0.90–0.98]*	excellent	1.05	excellent	0.19
TAIKT_6_ (n° kicks)	0.97 [0.92–0.99]*	excellent	0.68	excellent	0.15	1.00 [1.00–1.00]*	excellent	0.00	excellent	0.00
TAIKT_BEST_ (n° kicks)	0.97 [0.93–0.99]*	excellent	0.62	excellent	0.23	0.95 [0.89–0.98]*	excellent	0.98	excellent	0.19
TAIKT_TOTAL_ (n° kicks)	0.99 [0.98–1.00]*	excellent	0.48	excellent	0.44	1.00 [0.99–1.00]*	excellent	0.39	excellent	0.33
KDI (%)	0.87 [0.68–0.94]*	good	26.22	poor	1.33	0.79 [0.49–0.91]*	good	17.50	poor	1.49

FSKT_1_, FSKT_mult_ set 1; FSKT_2_, FSKT_mult_ set 2; FSKT_3_, FSKT_mult_ set 3; FSKT_4_, FSKT_mult_ set 4; FSKT_5_, FSKT_mult_ set 5; FSKT_BEST_, Set with the highest number of kicks in the FSKT_mult_; FSKT_TOTAL_, total number of kicks in the sets of FSKT_mult_; KDI, kick decrement index; TAIKT_1_, TAIKT_chest_ set 1; TAIKT_2_, TAIKT_chest_ set 2; TAIKT_3_, TAIKT_chest_ set 3; TAIKT_4_, TAIKT_chest_ set 4; TAIKT_5_, TAIKT_chest_ set 5; TAIKT_6_, TAIKT_chest_ set 6; TAIKT_BEST_, Set with the highest number of kicks in the TAIKT_chest_; TAIKT_TOTAL_, total number of kicks in the sets of TAIKT_chest_; ICC, intraclass correlation coefficient; CI, confidence intervals; CV, coefficient of variation. SEM, standard error of measurement; * = *p* < 0.001.

In addition, in order to express the tests’ performance also in terms of power, the method of Tayech et al., 2019; 2020; 2022 was used. The distances (*x*) and (*y*) allowed for determining the distance (*d*) using the Pythagorean theorem, which is the projection distance of the foot on the body protector. Thus, this distance (*d*) allowed for establishing the power of each kick set, as previously described (see (Tayech et al., 2019) and **Supplementary Material 1**). FSKT_mult_ and TAIKT_chest_ performances were expressed as absolute and relative (W and W·kg^-0.67^, respectively) peak and mean power (P_peak_ and P_mean_, respectively), and fatigue index (FI). Specifically, P_peak_: highest power output recorded during the sets of kicks; P_mean_: sum of powers recorded during the sets of kicks/number of sets; FI (%): [(P_peak_ − minimum power (P_min_)/P_peak_] × 100. The relative power of each kick set, using allometric scaling (Tayech et al., 2019; Tayech et al., 2020; Tayech et al., 2022), was calculated with the following formula: relative power = absolute power (W)/kg^0.67^.

**Physiological and Perceptual Measurements**. During the FSKT_mult_ and TAIKT_chest_, HR was beat-by-beat measured, using a Polar H10 monitor strap (Polar Electro Oy, Kempele, Finland), to record the following variables: mean heart rate (HR_MEAN_), which is the mean heart rate recorded during the test; peak heart rate (HR_PEAK_), which is the highest heart rate reached during the test. Furthermore, HR_PEAK_ was expressed as percentages of the athlete’s theoretical (208 − [0.7 × age]) maximal HR (%HR_max_) (Tanaka et al., 2001). [La] was recorded from the earlobe 3 minutes after the end of the FSKT_mult_ and TAIKT_chest_ using the Lactate Pro Analyzer (*Arkray, Tokyo, Japan*) (Tayech et al., 2019; Tayech et al., 2020; Tayech et al., 2022), which was calibrated before each measurement according to the manufacturer’s manual. RPE was recorded immediately (Tayech et al., 2019; Tayech et al., 2020; Tayech et al., 2022) after the end of high-intensity intermittent tests using the 15-point scale, which ranged from 6 (very, very light) to 20 (very, very hard) (Borg, 1982).

### 2.4 Statistical analyses

Data analyses were performed using Jamovi software (v. 2.6.45; The Jamovi Project, Australia). Average measures 2-way random ICC, with absolute agreement and 95%CI, was used to investigate the within-session relative reliability of CMJ test and intra-/inter-rater relative reliability of the FSKT_mult_ and TAIKT_chest_. The ICC values were interpreted as follows: <0.5: poor; 0.5–0.75: moderate; 0.76–0.9: good; >0.9: excellent (Koo and Li, 2016). While, CV and SEM were calculated as indicators of the absolute reliability. The CV values were interpreted as follows: <5%: excellent; <10%: very good; <15%: acceptable; >15%: poor (Hopkins et al., 2009). The Kolmogorov-Smirnov test revealed the normal distribution of all the considered variables. Therefore, data are presented as mean ± standard deviation and 95%CI. The relationships between the FSKT_mult_, TAIKT_chest_, and CMJ test variables were established using Pearson’s correlation coefficient (*r*) with 95%CI. The magnitude of correlations was assessed using the following benchmarks: 0–0.09: trivial; 0.1–0.29: low; 0.3–0.49: moderate; 0.5–0.69: large; 0.7–0.89: very large; ≥0.9: nearly perfect (Hopkins et al., 2009). Coefficient of determination (*R^2^*) was used to interpret the meaningfulness of the relationships. Convergent validity was accepted when a ‘‘large’’ value (or above) was observed between the FSKT_mult_ and TAIKT_chest_ (Tayech et al., 2022). To investigate whether prediction equations may be developed to determine the FSKT_mult_ performances from TAIKT_chest_ performances and vice versa, linear regression was used to model the relationship between the variables of these aforementioned two tests (Tayech et al., 2022). A paired t-test was used to compare variables between FSKT_mult_ and TAIKT_chest_. Cohen’s *d* with 95%CI was used as an effect size measure and was graded as: <0.2: trivial; 0.2–0.59: small; 0.6–1.19: moderate; 1.2–2.0: large; >2.0, very large (Hopkins et al., 2009). The statistical significance was accepted when *p* < 0.05.

## 3 Results

**Table 3** provides the correlation coefficient, confidence intervals, magnitude, and coefficient of determination of performances and physiological and perceptual measurements between FSKT_mult_ and TAIKT_chest_.

**Table 3:** Descriptive statistics and correlation coefficients of performances, and physiological and perceptual measurements between multiple frequency speed of kick test (FSKT_mult_) and chest taekwondo anaerobic intermittent kick test (TAIKT_chest_) (n = 22).

	FSKT_mult_	TAIKT_chest_				
	M ± SD [95%CI]	M ± SD [95%CI]	r [95%CI]	R^2^ (%)	Magnitude	p-value
TOTAL (n° kicks)	89 ± 6 [86–91]	57 ± 4 [55–59]	0.89 [0.75–0.95]	79	very large	< 0.001
KDI (%)	6 ± 3 [5–7]	4 ± 4 [2–5]	-0.11 [-0.51–0.32]	1	low	0.617
P_PEAK_ (W)	7 ± 2 [6–8]	16 ± 4 [14–17]	0.88 [0.73–0.95]	77	very large	< 0.001
P_PEAK_ (W.kg^-0.67^)	0.5 ± 0.1 [0.4–0.5]	1.0 ± 0.2 [0.9–1.1]	0.79 [0.55–0.91]	62	very large	< 0.001
P_MEAN_ (W)	6 ± 2 [6–7]	14 ± 3 [13–16]	0.96 [0.90–0.98]	92	nearly perfect	< 0.001
P_MEAN_ (W.kg^-0.67^)	0.4 ± 0.1 [0.4–0.4]	1.0 ± 0.2 [0.9–1.0]	0.91 [0.80–0.96]	83	nearly perfect	< 0.001
FI (%)	20 ± 9 [16–24]	13 ± 11 [8–17]	-0.12 [-0.52–0.32]	1	low	0.599
HR_MEAN_ (b.min^-1^)	181 ± 7 [178–184]	177 ± 9 [173–181]	0.87 [0.71–0.95]	76	very large	< 0.001
HR_PEAK_ (b.min^-1^)	187 ± 7 [184–190]	184 ± 9 [180–188]	0.92 [0.82–0.96]	85	nearly perfect	< 0.001
%HR_MAX_	95 ± 4 [94–97]	94 ± 5 [92–96]	0.90 [0.77–0.96]	81	very large	< 0.001
[La] (mmol.l^-1^)	12 ± 3 [11–13]	10 ± 2 [9–11]	0.61 [0.25–0.82]	37	large	0.003
RPE (a.u.)	16 ± 2 [15–16]	14 ± 1 [13–15]	0.68 [0.36–0.85]	46	large	0.001

TOTAL, total number of kicks in the test sets; KDI, kick decrement index; P_PEAK_, peak power; P_MEAN_, mean power; FI, fatigue index; HR_MEAN_, mean heart rate; HR_PEAK_, peak heart rate; %HR_MAX_, peak HR expressed as percentages of the athlete’s theoretical maximal HR; [La], blood lactate concentration; RPE, rating of perceived exertion scale; M, mean; SD, standard deviation; CI, confidence intervals; r, Pearson’s coefficient; R^2^, coefficient of determination.

Total kick performance (i.e., TOTAL), as well as absolute and relative peak and mean (i.e., P_peak_ and P_mean_) power performances, showed high significant correlations (from “very large” to “nearly perfect”). In contrast, fatigue-related indexes (i.e., KDI and FI) revealed no significant correlations. All physiological and perceptual measurements (i.e., HR, [La], and RPE) showed high significant correlations (from “large” to “nearly perfect”).

**Table 4** presents the resulting regression equations to estimate FSKT_mult_ from TAIKT_chest_ performances and vice versa.

**Table 4:** Regression equations to estimate multiple frequency speed of kick test (FSKT_mult_) from chest taekwondo anaerobic intermittent kick test (TAIKT_chest_) performances and vice versa (n = 22).

FSKT_mult_	TAIKT_chest_	*R^2^*	Adjusted *R^2^*	SEE	*p*-value
TOTAL (n° kicks)	19.83 + 1.20 (TOTALn°kicks)	0.79	0.78	2.86	< 0.001
P_PEAK_ (W)	0.28 + 0.44 (P_PEAK_W)	0.77	0.76	0.92	< 0.001
P_PEAK_ (W.kg^-0.67^)	0.08 + 0.37 (P_PEAK_W.kg^-0.67^)	0.62	0.60	0.06	< 0.001
P_MEAN_ (W)	0.07 + 0.43 (P_MEAN_W)	0.92	0.91	0.44	< 0.001
P_MEAN_ (W.kg^-0.67^)	0.04 + 0.39 (P_MEAN_W.kg^-0.67^)	0.83	0.82	0.03	< 0.001
TAIKT_chest_	FSKT_mult_	*R^2^*	Adjusted *R^2^*	SEE	*p*-value
TOTAL (n° kicks)	-0.89 + 0.66 (TOTALn°kicks)	0.79	0.78	2.12	< 0.001
P_PEAK_ (W)	3.03 + 1.76 (P_PEAK_W)	0.77	0.76	1.85	< 0.001
P_PEAK_ (W.kg^-0.67^)	0.25 + 1.66 (P_PEAK_W.kg^-0.67^)	0.62	0.60	0.12	< 0.001
P_MEAN_ (W)	1.05 + 2.15 (P_MEAN_W)	0.92	0.91	1.00	< 0.001
P_MEAN_ (W.kg^-0.67^)	0.07 + 2.16 (P_MEAN_W.kg^-0.67^)	0.83	0.82	0.07	< 0.001

TOTAL, total number of kicks in the test sets; P_PEAK_, peak power; P_MEAN_, mean power; *R^2^*, coefficient of determination; SEE, standard error of estimate.

**Figure 2:**
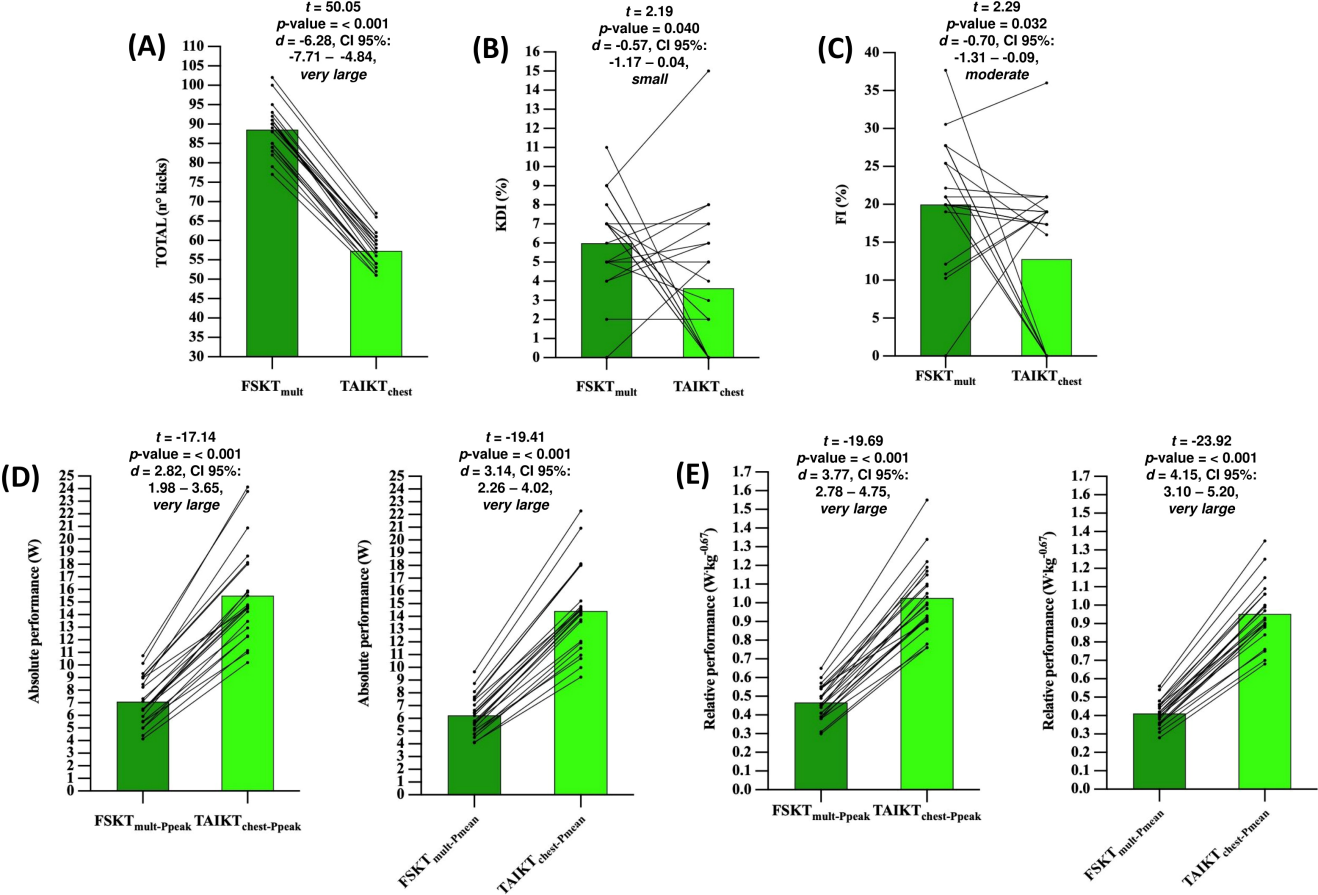
Comparison of performances between multiple frequency speed of kick test (FSKT_mult_) and chest taekwondo anaerobic intermittent kick test (TAIKT_chest_) (n = 22). **(A)** Comparison of total kicks; **(B)** Comparison of kick decrement index (KDI); **(C)** Comparison of fatigue index (FI); **(D)** Comparison of absolute peak and mean powers; **(E)** Comparison of relative peak and mean powers. *t*, t-test; *d*, Cohen’s *d*; CI, confidence intervals.

**Figure 3:**
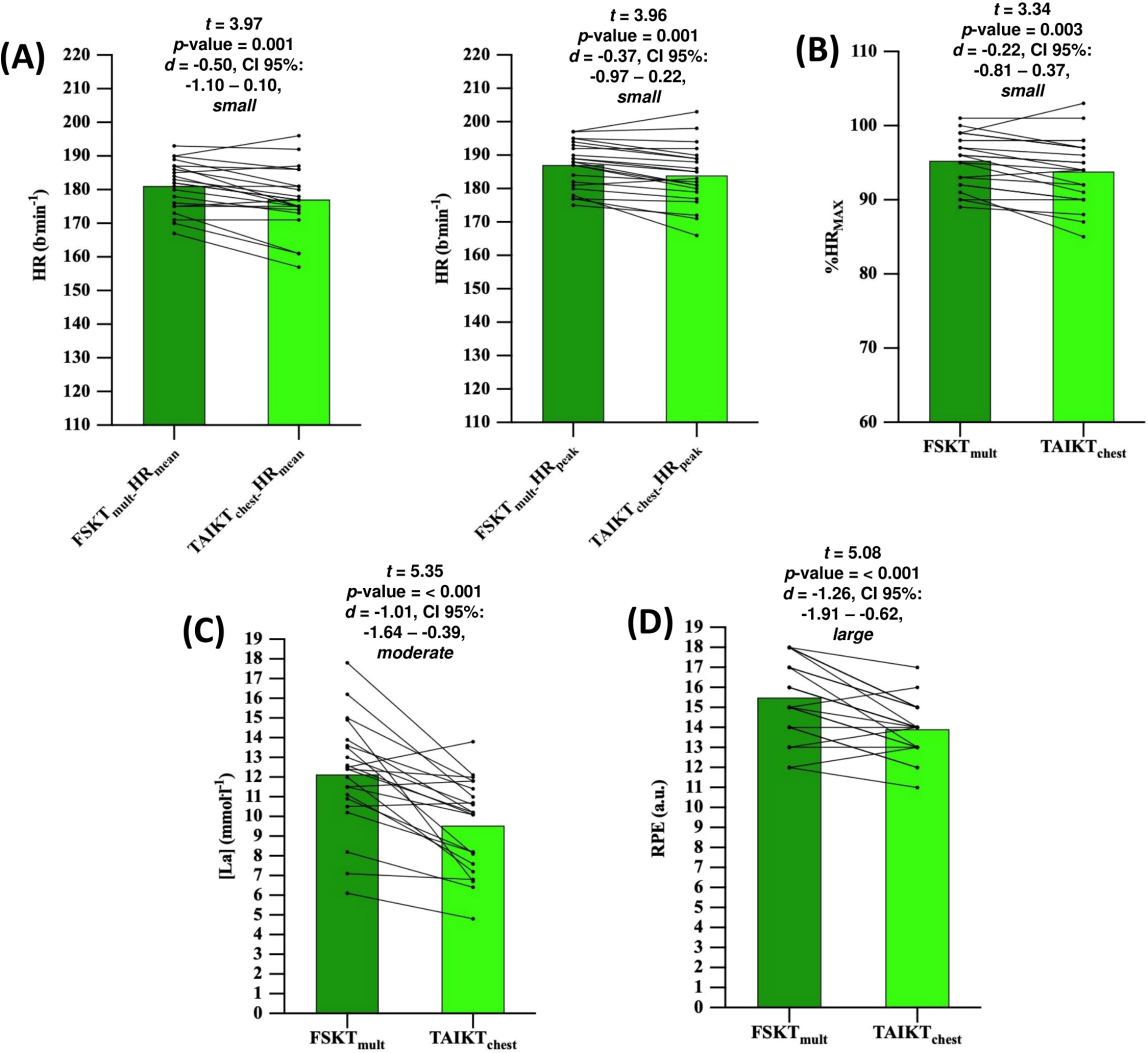
Comparison of physiological and perceptual measurements between multiple frequency speed of kick test (FSKT_mult_) and chest taekwondo anaerobic intermittent kick test (TAIKT_chest_) (n = 22). **(A)** Comparison of mean and peak heart rate (HR); **(B)** Comparison of peak HR expressed as percentages of the athlete’s theoretical maximal HR (%HR_MAX_); **(C)** Comparison of blood lactate [La] concentration; **(D)** Comparison of rating of perceived exertion (RPE) scale. *t*, t-test; *d*, Cohen’s *d*; CI, confidence intervals.

**Figures 2**, **3** show the comparison of performances and physiological and perceptual measurements between FSKT_mult_ and TAIKT_chest_.

Total kick performance (i.e., TOTAL), as well as fatigue-related indexes (i.e., KDI and FI), were significantly higher (from “small” to “very large”) in FSKT_mult_ than in TAIKT_chest_. In contrast, absolute and relative peak and mean power performances (i.e., P_peak_ and P_mean_), were significantly higher (“very large”) in TAIKT_chest_ than in FSKT_mult_. All physiological and perceptual measurements (i.e., HR, [La], and RPE) were significantly higher (from “small” to “large”) in the FSKT_mult_ than in TAIKT_chest_.

**Table 5** shows the correlation coefficient, confidence intervals, magnitude, and coefficient of determination of performances between FSKT_mult_ and TAIKT_chest_, and CMJ test.

**Table 5:** Correlation coefficients of performances between multiple frequency speed of kick test (FSKT_mult_) and chest taekwondo anaerobic intermittent kick test (TAIKT_chest_), and countermovement jump (CMJ) test (n = 22).

	FSKT_TOTAL_ (n° kicks)	FSKT_mult-Ppeak_ (W)	FSKT_mult-Ppeak_ (W.kg^-0.67^)	TAIKT_TOTAL_ (n° kicks)	TAIKT_chest-Ppeak_ (W)	TAIKT_chest-Ppeak_ (W.kg^-0.67^)
CMJ_height_ (cm) *r* [95%CI] *R^2^* (%) *Magnitude* *p*-value	0.60 [0.24–0.82] 36 *large* 0.003	–	–	0.37 [-0.06–0.69] 14 *moderate* 0.088	–	–
CMJ_Ppeak_ (W) *r* [95%CI] *R^2^* (%) *Magnitude* *p*-value	–	0.72 [0.44–0.88] 53 *very large* < 0.001	–	–	0.58 [0.22–0.81] 34 *large* 0.004	–
CMJ_Ppeak_ (W.kg^-0.67^) *r* [95%CI] *R^2^* (%) *Magnitude* *p*-value	–	–	0.51 [0.12–0.76] 26 *large* 0.016	–	–	0.30 [-0.15–0.64] 9 *low* 0.183
Net impulse (N.s) *r* [95%CI] *R^2^* (%) *Magnitude* *p*-value	0.31 [-0.13–0.65] 10 *moderate* 0.158	0.78 [0.53–0.90] 61 *very large* < 0.001	. 0.66 [0.32–0.84] 44 *large* < 0.001	. 0.07 [-0.36–0.48] 1 *trivial* 0.746	0.64 [0.30–0.84] 41 *large* 0.001	0.40 [-0.03–0.70] 16 *moderate* 0.066

FSKT_TOTAL_, total number of kicks in the sets of FSKT_mult_; FSKT_mult-Ppeak_, peak power during FSKT_mult_; TAIKT_TOTAL_, total number of kicks in the sets of TAIKT_chest_; TAIKT_chest-Ppeak_, peak power during TAIKT_chest_; CMJ_height_, height of CMJ test; CMJ_Ppeak_, peak power of CMJ test; *r*, Pearson’s coefficient; CI, confidence intervals; *R^2^*, coefficient of determination.

CMJ_height_, absolute CMJ_Ppeak_, relative CMJ_Ppeak_, and net impulse were 32.4 ± 7.5 [29.1–35.7] cm, 2507 ± 647 [2220–2794] W, 165 ± 33 [151–180] W.kg^-0.67^, and 144 ± 29 [131–157] N.s, respectively. FSKT_mult_ performances (i.e., FSKT_TOTAL_, absolute and relative FSKT_mult-Ppeak_) showed high significant correlations (from “large” to “very large”) with respective CMJ performances (i.e., CMJ_height_, absolute and relative CMJ_Ppeak_). Furthermore, the absolute and relative peak power performances (i.e., FSKT_mult-Ppeak_) showed high significant correlations (from “large” to “very large”) with net impulse. In contrast, only the absolute peak power performance (i.e., TAIKT_chest-Ppeak_) of TAIKT_chest_ was highly correlated (“large”) with respective CMJ performance (i.e., absolute CMJ_Ppeak_) and net impulse.

## 4 Discussion

The first aim of this study was to establish the convergent validity between the FSKT_mult_ and TAIKT_chest_, which are the two most studied and used sport-specific tests in practice to assess high-intensity intermittent performance in taekwondo. The hypothesis was confirmed as positive correlations from large to nearly perfect emerged between the tests, both for performances (i.e., total kick, absolute and relative peak and mean power) and physiological and perceptual measurements (i.e., HR, [La], and RPE). However, fatigue-related indexes (i.e., KDI and FI) did not reveal significant correlations. Furthermore, all performances and physiological and perceptual measurements differed significantly between tests. The second aim was to investigate the relationship between the above sport-specific tests and the CMJ, which is the test commonly used to assess lower limb muscle power among taekwondo athletes. The hypothesis was confirmed, as positive large or very large relationships were found between most high-intensity intermittent performances (i.e., total kick, absolute and relative peak power) and the respective lower limb muscle power performances (i.e., height reached, absolute and relative peak power).

In line with the literature (Apollaro et al., 2024b; Apollaro et al., 2024c; Apollaro et al., 2025), the post-test video analysis procedure to count the number of valid techniques revealed an excellent intra-/inter-rater reliability for the FSKT_mult_ performances, except for the KDI. The peculiarity of this index formula is that a minimal possible difference (such as a single kick) in the best set between the two counts increases the denominator of the KDI formula (i.e. the theoretical maximum) more than the sum of the sets in the numerator between the two counts can generally increase in parallel. As a result, this mathematical property of the formula may amplify the difference in kick decrement between the two counts and the resulting reliability statistics (for a practical example, see the **Supplementary Material 2**). In this regard, the acceptable absolute intra-/inter-rater reliability of the KDI (i.e., CV~10%) is consistent with previous findings (Apollaro et al., 2024c; Apollaro et al., 2025). In parallel, this study was the first to extend the use of the traditional body protector to the TAIKT_chest_, and the resulting technique counting procedure typical of the FSKT_mult_, thus reducing the cost of the assessment. Indeed, it is worth noting that, in the original TAIKT_chest_ protocol, valid kicks are automatically recorded by an electronic body protector. An excellent intra-/inter-rater reliability also emerged for the TAIKT_chest_ performances, except for the KDI. In particular, the poor intra-/inter-rater reliability of the KDI (i.e., CV>15%) confirms the insights into this index, logically indicating that the shorter duration of the TAIKT_chest_ sets (and the consequent fewer kicks than the FSKT_mult_) accentuates the difference of the kick decrement between the two counts.

The FSKT_mult_ and TAIKT_chest_ showed high shared variance for both performances (i.e., *R*^2^ = 62–92%) and physiological and perceptual measurements (i.e., *R*^2^ = 37–85%); thus, providing their convergent validity. In this sense, the resulting regression equations were developed to allow coaches and strength and conditioning professionals to estimate FSKT_mult_ performances from those of TAIKT_chest_ and vice versa. However, the low correlations between tests for fatigue-related indexes alone (i.e., KDI and FI) suggest that the distinct methodological characteristics (in particular, single and total attack phase duration, and A/S ratio) generate highly specific kick decrement dynamics. In fact, the TAIKT_chest_ has six shorter all-out attack sets (i.e., 5 s and close to those of the ~2 s match (Bridge et al., 2011; Santos et al., 2011; Bridge et al., 2013; Apollaro et al., 2023; Apollaro et al., 2025)) interspersed by active recovery phases of double duration that support the maintenance of performance from set to set, as also justified by the significantly lower KDI and FI compared to those of the FSKT_mult_. In contrast, the FSKT_mult_ has five longer all-out attack sets (i.e., 10s) interspersed by passive recovery phases of the same duration, which result in a greater decrease of performances. Recently, Tayech et al. (2022) developed the head TAIKT (TAIKT_head_), which is a test with the same methodological and measurement characteristics as the TAIKT_chest_, but with the kicking technique (i.e., bandal-chagi) projected at the head of the dummy, protected by an electronic head protector. In this context, it is interesting to note that the fatigue index (i.e., FI) correlated highly between the TAIKT_chest_ and the TAIKT_head_, thus confirming the impact of the single and total attack phase duration and the A/S ratio on the decrease of performances (Tayech et al., 2022).

In addition to significantly lower KDI and FI in TAIKT_chest_ compared to FSKT_mult_, all performances differed significantly between tests. The lower total kick performance in the TAIKT_chest_ reveals a logically expected result due to the shorter duration of the sets compared to those of the FSKT_mult_. Despite this, the comparison between performances is fundamental in highlighting that, on the contrary, performances expressed in terms of power (i.e., absolute and relative peak and mean power) were higher in the TAIKT_chest_. In the present study, we extended to the FSKT_mult_ the calculation method specifically developed to express TAIKT_chest_ performance in terms of power (Tayech et al., 2019), thus increasing the information that can be obtained from the test. The differences in power performances are justified by the fact that, in the formula used, the number of kicks in the numerator increases quadratically, while the time in the denominator increases cubically. Thus, assuming almost twice as many kicks in each set of the FSKT_mult_ compared to the TAIKT_chest_, the duration of the sets continues to be responsible for the lower power expressed in the FSKT_mult_. This suggests the future need to specifically adapt the formula to the characteristics of the FSKT_mult_, thus allowing for consistent comparison between tests. In addition, all physiological and perceptual responses (i.e., HR, [La], and RPE) were significantly higher in the FSKT_mult_, further corroborating the impact of distinct methodological characteristics. However, it is noteworthy that both tests elicited physiological and perceptual responses fairly close to those of official matches documented in the literature (i.e., %HR_MAX_: ~96–97%; [La]: 6.7–14.0 mmol L^-1^; RPE: ~14 a.u.) (Bridge et al., 2014; Santos et al., 2020; Apollaro et al., 2024a; Apollaro et al., 2024b).

In line with some studies (Albuquerque et al., 2022; Apollaro et al., 2024c), CMJ height, as well as the absolute and relative peak power explained 36%, 53% and 26% of the variance of the respective FSKT_mult_ performances. Furthermore, the net impulse explained 61% and 44% of the variance of the absolute and relative peak power of the FSKT_mult_, respectively. In the present study, the availability of two tests with distinct methodological characteristics allowed to expand the study of the relationship between the ability to repeat high-intensity kicks and the lower limb muscle power. In this sense, it is relevant to highlight that the absolute peak power and net impulse of the CMJ explained 34% and 41% of the variance of the absolute peak power of the TAIKT_chest_, while the height reached and the relative peak power did not correlate significantly with the respective performances. The findings of the present study suggest that the highest performance (i.e., P_peak_) in the FSKT_mult_ and TAIKT_chest_, generally achieved in set 1 or 2, depends in part on lower limb muscle power.

### 4.1 Limitations and future directions

It is important to recognize the limitation of this study related to the inclusion of black belt only athletes of national/international level. Previously, the study of the discriminant validity of the two tests revealed differences in performances between athletes of different competitive levels (i.e., international-/national *vs.* state-/regional or elite *vs.* sub-elite) (Apollaro et al., 2024b). Therefore, the study of convergent validity in athletes of other competitive levels would help determine whether the results presented here also apply to those populations. On the other hand, the convergent validity between the FSKT_mult_ and TAIKT_chest_ emphasized the peculiarities of each test in relation to physiological and perceptual responses, thus suggesting the study of translational content validity (i.e., the extent that the content of a test matches and measures all elements of a given construct (Weakley et al., 2024)) as the next fundamental research step. Indeed, considering the distinct physiological and perceptual responses, the estimation of the energy systems’ contribution (i.e., ATP-PCr, glycolytic, and oxidative) would directly provide the content validity of each intermittent sport-specific test (Würdig et al., 2023; Apollaro et al., 2024b).

### 4.2 Practical applications

Both the FSKT_mult_ and the TAIKT_chest_ can be used to monitor high-intensity intermittent performance in sport-specific settings. In this sense, based on the test used, the equations developed in this study allow to extrapolate the respective performances of the other test, gaining more insights from a single assessment. Furthermore, the excellent intra-/inter-rater reliability for the performances of both tests supports the use of traditional body protector, greatly reducing the cost of assessment. In parallel, the method for expressing performances in terms of power can be easily applied to both tests, increasing the information that can be obtained. Lower limb muscle power should be specifically trained to emphasize peak performance during intermittent sport-specific activity.

## 5 Conclusions

The FSKT_mult_ and TAIKT_chest_ showed high shared variance for both performances and physiological and perceptual measurements; thus, proving their convergent validity. However, the distinct methodological characteristics of the tests such as the duration of each set and the entire test, as well as the attack/skipping ratio, generate highly specific performance, physiological, and perceptual dynamics. Finally, greater lower limb muscle power allows for higher peak performance during intermittent sport-specific activity.

## Data Availability

The original contributions presented in the study are included in the article/**Supplementary Material**. Further inquiries can be directed to the corresponding author.
