# Supplementary material for: Convergent validity of taekwondo high-intensity intermittent sport-specific tests and their relationship with lower limb muscle power performance
Source: Front Physiol. 2026 Jun 1;17:1825858. doi: 10.3389/fphys.2026.1825858 (PMC13267583; doi:10.3389/fphys.2026.1825858)
Supplement: Supplementary file 1 [file Table1.docx]

The power of each kick set of the Multiple Frequency Speed of Kick Test (FSKT_mult_) and Chest Taekwondo Anaerobic Intermittent Kick Test (TAIKT_chest_) was determined based on this formula, as previously described by Tayech et al. (2):

Power (W) = MLL × (d × N_kicks_)^2^ / (10_s_ or 5_s_)^3^ 🡪 Power (W) = Force × Velocity

Force:

- Force = mass of the lower limb (MLL) (kg) × acceleration 🡪 (Force = mass × acceleration)

🡺 Acceleration = velocity / 10_s_ or 5_s_ 🡪 Acceleration = velocity (m·s^-1^) / time (s)

NB. 10_s_ and 5_s_ represent the execution time (s) for each kick set of the FSKT_mult_ and TAIKT_chest_, respectively.

🡺 Thereby

- Force = mass of the lower limb (MLL) (kg) × (velocity / 10_s_ or 5_s_)

Velocity:

- Velocity = [d × (number of kicks (N_kicks_))] / 10_s_ or 5_s_ 🡪 Velocity = distance / time (s)

NB. d × N_kicks_ is the total distance (m) of kicks set.

NB. 10_s_ and 5_s_ represent the execution time (s) for each kicks set of the FSKT_mult_ and TAIKT_chest_, respectively.

Power:

Power (W) = Force × Velocity 🡪

Power (W) = (MLL (kg) × (velocity / 10_s_ or 5_s_)) × ([d × (N_kicks_)] / 10_s_ or 5_s_) 🡪

Power (W) = (MLL (kg) × ((d × N_kicks_) / 10_s_ or 5_s_) / 10_s_ or 5_s_)) × ([d × (N_kicks_)] / 10_s_ or 5_s_) 🡪

finally 🡪

Power (W) = MLL × (d × N_kicks_)^2^ / (10_s_ or 5_s_)^3^

NB. MLL is the mass of the lower limb (kg)

NB. The MLL calculation was based on the method previously described by Hamill et al. (1):

- Thigh weight (N): 0.127(BW) – 14.82

- Leg weight (N): 0.044(BW) – 1.75

- Foot weight (N): 0.009(BW) – 2.48

- Add thigh, leg and foot weights to get weight of the lower limb.
- Body weight (BW) is in Newton (N)
- Convert Newton to Kilogram (1kg = 9.806 N)

**References:**

1. Hamill J., Knutzen K. M., Derrick T. R. *Biomechanical Basis of Human Movement*, 4th, Philadelphia, PA: Lippincott Williams & Wilkins, a Wolters Kluwer business, 2015.
2. Tayech A., Mejri M. A., Chaabene H., Chaouachi M., Behm D. G., Chaouachi A. (2019). Test-retest reliability and criterion validity of a new Taekwondo Anaerobic Intermittent Kick Test. *J. Sports Med. Phys Fitness.* 59(2), 230–237. doi:10.23736/S0022-4707.18.08105-7
