# Supplementary material for: Convergent validity of taekwondo high-intensity intermittent sport-specific tests and their relationship with lower limb muscle power performance
Source: Front Physiol. 2026 Jun 1;17:1825858. doi: 10.3389/fphys.2026.1825858 (PMC13267583; doi:10.3389/fphys.2026.1825858)
Supplement: Supplementary file 2 [file Table2.docx]

- Kick decrement index (KDI) equation (1,2):

$$KDI \left( \% \right)=\left[ 1- \frac{\mathrm{set}1 + set2 + set3 +..}{best set \times number of sets} \right]\times100$$

Theoretical maximum

**Example of intra-rater reliability for the FSKT_mult_**:

First count: Second count:

$KDI \left( \% \right)=\left[ 1-\frac{17+16+16+15+15}{17 \times5} \right]\times100$ $KDI \left( \% \right)=\left[ 1-\frac{18+16+16+15+15}{18 \times5} \right]\times100$

Increase of 1 kick between counts in the best set.

The numerator increases by 1 kick.

$KDI \left( \% \right)=\left[ 1-\frac{79}{85} \right]\times100$ $KDI \left( \% \right)=\left[ 1-\frac{80}{90} \right]\times100$

The theoretical maximum increases by 5 kicks.

$KDI \left( \% \right)=\left[ 1-0,93 \right]\times100= \boldsymbol{7\%}$ $KDI \left( \% \right)=\left[ 1-0,89 \right] \times100= \mathbf{11}$**%**

The increase of 1 kick in the best set results in a 4 percentage point increase in the KDI.

**References:**

1. Apollaro G., Ouergui I., Rodríguez Y. Q., Kons R. L., Detanico D., Franchini E., et al. (2024b). Anaerobic sport-specific tests for taekwondo: A narrative review with guidelines for the assessment. *Sports* 12 (10), 278. [doi:10.3390/sports12100278](https://doi.org/10.3390/sports12100278)
2. da Silva Santos J. F., Lopes-Silva J. P., Loturco I., Franchini E. (2020). Test-retest reliability, sensibility and construct validity of the frequency speed of kick test in male black-belt Taekwondo athletes. *Ido Mov. Cult.* 20(3), 38–46. doi:10.14589/ido.20.3.6
